# Veterinary students’ choices of clinical extramural placements are associated with their final‐year examination performance

**DOI:** 10.1002/vetr.70553

**Published:** 2026-04-01

**Authors:** Matthew Barden, Emma Ormandy, Melanie Chapman, Alistair Freeman, Kieron Salmon

**Affiliations:** ^1^ School of Veterinary Science Institute of Infection Veterinary and Ecological Sciences University of Liverpool Liverpool UK

## Abstract

**Background:**

UK veterinary students must complete 20 weeks of clinical extramural studies (EMS) in placements of their choice. We investigated the associations between EMS choices and performance in the final‐year ‘livestock and One Health’, ‘equine’ and ‘small animal’ written examinations.

**Methods:**

This retrospective cohort study utilised data from 639 students who took their final examinations between 2020 and 2024 at the University of Liverpool. The mark (%) and outcome (passed/failed) of each examination were analysed in separate regression models with the number of clinical EMS weeks in each discipline (farm animal/public health, equine, small animal or mixed practice) as explanatory variables. Overall pass rates were compared between students with and without balanced EMS, defined as 2 or more weeks in each species or 8 or more weeks in mixed practice.

**Results:**

An increased number of EMS weeks in the corresponding species or in mixed practice was associated with improved examination marks. Among students with weaker preclinical performance, those with balanced EMS experience were more likely to pass all their final examinations.

**Limitations:**

This study was conducted at a single UK veterinary school and relied on self‐reporting of EMS type.

**Conclusion:**

Clinical EMS is associated with final‐year examination performance, although causality cannot be inferred.

## INTRODUCTION

Extramural studies (EMS) are mandatory for all veterinary students in the UK and aim to develop clinical and professional skills through workplace‐based experiential learning. Survey data suggest that veterinary students and recent graduates find EMS to be a valuable component of their training, while also identifying areas for improvement.^1^, ^2^, ^3^

From 2024 onwards, the Royal College of Veterinary Surgeons (RCVS) requires a minimum of 30 weeks of EMS for students, comprising 10 weeks of animal husbandry EMS and 20 weeks of clinical EMS. This represents a reduction from the previous requirements of 12 weeks of animal husbandry EMS and 26 weeks of clinical EMS, which applied to students starting before 2024. Under these new regulations, the RCVS prevents universities from imposing species‐specific quotas on placements, affording students the agency to tailor placements to their interests or learning needs.^4^ Career aspirations and practical factors (e.g., cost) are reported as the most influential factors in students’ EMS choices.^3^

In the Bachelor of Veterinary Science programme at the University of Liverpool, students study predominantly preclinical subjects in years 1‒3 and clinical subjects in years 4 and 5. At the end of year 5, students must pass three written examinations: ‘livestock and One Health (LOH)’, ‘equine’ and ‘small animal’. The LOH examination comprises single best answer (SBA) questions and short written responses to clinical scenarios, while the equine and small animal examinations consist solely of SBA questions. All the examinations assess clinical reasoning and applied knowledge across the cognitive domain.

As part of the University of Liverpool's student success framework, all students have an academic adviser.^5^, ^6^ Advisers meet with students at least twice a year during professional development planning meetings to offer academic guidance and support, including advice on EMS planning. Whether EMS placements influence examination performance remains unclear, limiting academic advisers’ ability to provide evidence‐based guidance on EMS planning.

This study aimed to evaluate the association between type and number of clinical EMS placements and final‐year examination performance in the LOH, equine and small animal examinations. We considered examination performance from three perspectives: the mark in each examination, whether the student passed or failed each examination, and whether the student passed all three final‐year examinations.

## MATERIALS AND METHODS

### Study design

This retrospective cohort study was conducted and reported following the Strengthening the Reporting of Observational Studies in Epidemiology guidelines.^7^ This research was undertaken as part of the Postgraduate Certificate in Academic Practice programme.^8^ Ethical approval for research projects conducted as part of this programme was granted by the University of Liverpool Professional Services Research Ethics Committee (REF 14008).

### Data source and editing

We compiled a dataset of pseudonymised records of EMS placements and examination results for Bachelor of Veterinary Science students at the University of Liverpool. All available data for 639 students who took their final written examinations between 2020 and 2024 were included (2020: *n* = 124; 2021: *n* = 121; 2022: *n* = 134; 2023: *n* = 127; 2024: *n* = 133), with no additional eligibility criteria.

COVID‐19 impacted the study cohort. The 2020 cohort experienced open‐book, online examinations without invigilation. The 2021 cohort underwent online examinations. From 2022 onwards, examinations returned to an in‐person format. Examination results were collated from first attempts after they had been standard set using a modified Hofstee method and normalised to a 50% pass mark.^9^

The standard RCVS requirement was 26 weeks of clinical EMS, with the University of Liverpool policy additionally requiring a minimum of 2 weeks in each of small animal, equine and farm animal practice. In response to COVID‐19, the RCVS temporarily reduced the minimum clinical EMS requirements, varying by graduation year: 13 weeks for students graduating in 2021‒2022, 17 weeks for students graduating in 2023 and 20 weeks for students graduating in 2024. Species‐specific requirements were suspended during the pandemic and subsequently removed from the University of Liverpool policy in early 2022. Because students may complete clinical EMS at any point in their final 3 years, the precise policy requirements applicable to individual students varied depending on when they undertook their placements. Consequently, students graduating in 2020 were more likely to have completed EMS under the original species‐specific policy.

Clinical EMS placements were categorised based on student‐reported descriptions as either ‘small animal’, ‘equine’, ‘farm animal’, ‘mixed’, ‘other’, ‘public health’ or ‘research’. Weeks of farm animal and public health placements were combined into a single category, referred to as LOH, to reflect the examination structure. The number of completed weeks (including partial weeks) for each placement category and each student was recorded. Placements labelled as ‘other’ or ‘research’ were excluded from further analysis due to their non‐clinical focus or variability. All the students had complete data for examination outcomes and EMS records. Preclinical marks were available for all students.

### Variables

Three outcomes related to examination performance were investigated: (1) examination mark (0%‒100%), (2) examination success (passed or failed) and (3) overall examination success (passed all three examinations or failed at least one examination). The explanatory variables of interest were the number of weeks in each EMS category (continuous variables). Additional covariates included the student's average preclinical mark (the mean of years 1‒3 examination marks, excluding practical examinations) and the year of the final‐year examination (a categorical variable).

### Statistical methods

All analyses were conducted in R version 4.4.2 (R Core Team, 2021) using tidyverse packages, unless otherwise stated.^10^

#### Outcome 1: Examination mark

We investigated whether the number of weeks spent in each EMS placement category (small animal, equine, LOH and mixed) was associated with students’ percentage scores in the small animal, equine and LOH examinations. We fitted three linear regression models, with each examination mark as the dependent variable and the number of weeks for each EMS type, the average preclinical examination mark and the year of examination as independent variables. All EMS terms were retained in the models regardless of statistical significance to allow direct comparison between models.

Because the species composition of mixed EMS placements was unknown and likely overlapped with that of the species‐specific EMS, we tested whether a shared slope could capture their combined effects on examination marks. For each model, we tested whether the slope for the corresponding species‐specific EMS weeks (e.g., small animal EMS weeks for the small animal examination model) was equivalent to the slope for mixed EMS weeks using a Wald test.^11^ Where slope equivalence was not rejected (*p* > 0.05), the terms were replaced by a single combined variable (i.e., the sum of the species‐specific and mixed EMS weeks). The separate‐term and simpler models were compared using an *F*‐test and the Akaike information criterion.

Two‐way interaction terms between the main EMS variables of interest and the average preclinical examination mark and year‐of‐examination terms were tested by adding each interaction term individually to the additive model. Significance was assessed using likelihood ratio tests. Interactions with a *p*‐value of less than 0.05 were retained in the final models.

The year of examination was included as a fixed effect rather than a random effect because (i) only 5 year groups were available, providing a limited basis for random‐effects variance estimation; (ii) we aimed to estimate and interpret year‐specific effects given potential COVID‐19 impacts and changes to EMS policy; and (iii) fixed effects permitted testing for interactions between year and EMS variables.

Model assumptions were evaluated using residuals versus fitted values, scale‐location plots, *Q*‒*Q* plots, and residuals versus leverage plots. Interaction effects and predicted examination marks were visualised using model‐based predictions over a range of EMS weeks and covariate levels.

#### Outcome 2: Examination success

We investigated whether the number of weeks spent in each species‐specific EMS category (LOH, equine and small animal) was associated with the binary outcome of passing the final‐year examinations (LOH, equine and small animal).

Because of the small number of examination failures in the dataset (20/639 for LOH, 10/639 for equine and 28/639 for small animal), we took a more limited modelling approach for this outcome. We fitted separate logistic regression models for each examination outcome using Firth's penalised logistic regression using the logistf package to obtain more reliable coefficient estimates and confidence intervals in the context of sparse data.^12^ The primary predictor of interest was the number of weeks in the corresponding EMS category. Models included year and preclinical mark as covariates.

#### Outcome 3: Overall examination success

Finally, we investigated whether a balanced approach to clinical EMS was associated with students passing all their final‐year examinations (‘overall examination success’). A balanced EMS experience was defined as completing at least 2 weeks each of small animal, equine and LOH EMS placements, in line with a previous University of Liverpool EMS policy. We also evaluated whether completing 6 or 8 weeks of mixed EMS might offer a comparable alternative to providing a balanced EMS experience.

Exploratory analysis indicated that students with an average preclinical mark below 60% (*n* = 106) were more likely to fail a final‐year examination. We therefore focused this exploratory analysis on the subset of students who appeared to be at greater risk of examination failure, based on a visual inspection of the data. We generated 2 × 2 contingency tables, with one binary variable indicating whether the student passed all three final‐year examinations and the other indicating whether the student had a balanced EMS experience (defined in three ways): (i) ≥2 weeks in each of small animal, equine and LOH; (ii) the previous criteria or ≥6 weeks of mixed EMS; and (iii) the previous criteria or ≥8 weeks of mixed EMS). Associations were tested using Fisher's exact tests.

## RESULTS

Records from 639 students were included in the dataset. The distribution of weeks spent in different types of clinical EMS is shown in Figure 1. Students completed a median of 19.2 weeks (interquartile range [IQR] 15.2–27.2) of clinical EMS, with variation between years reflecting changes in regulatory requirements (2020: 26.0; 2021: 16.8; 2022: 15.0; 2023: 16.0; 2024: 20.2). The median number of weeks of the different types of clinical EMS completed by students was 9.0 (IQR 6.0–12.0) in small animal, 2.6 (IQR 2.0–4.9) in equine, 3.0 (IQR 2.0–5.0) in LOH and 0.4 (IQR 0.0–3.0) in mixed.

**FIGURE 1:**
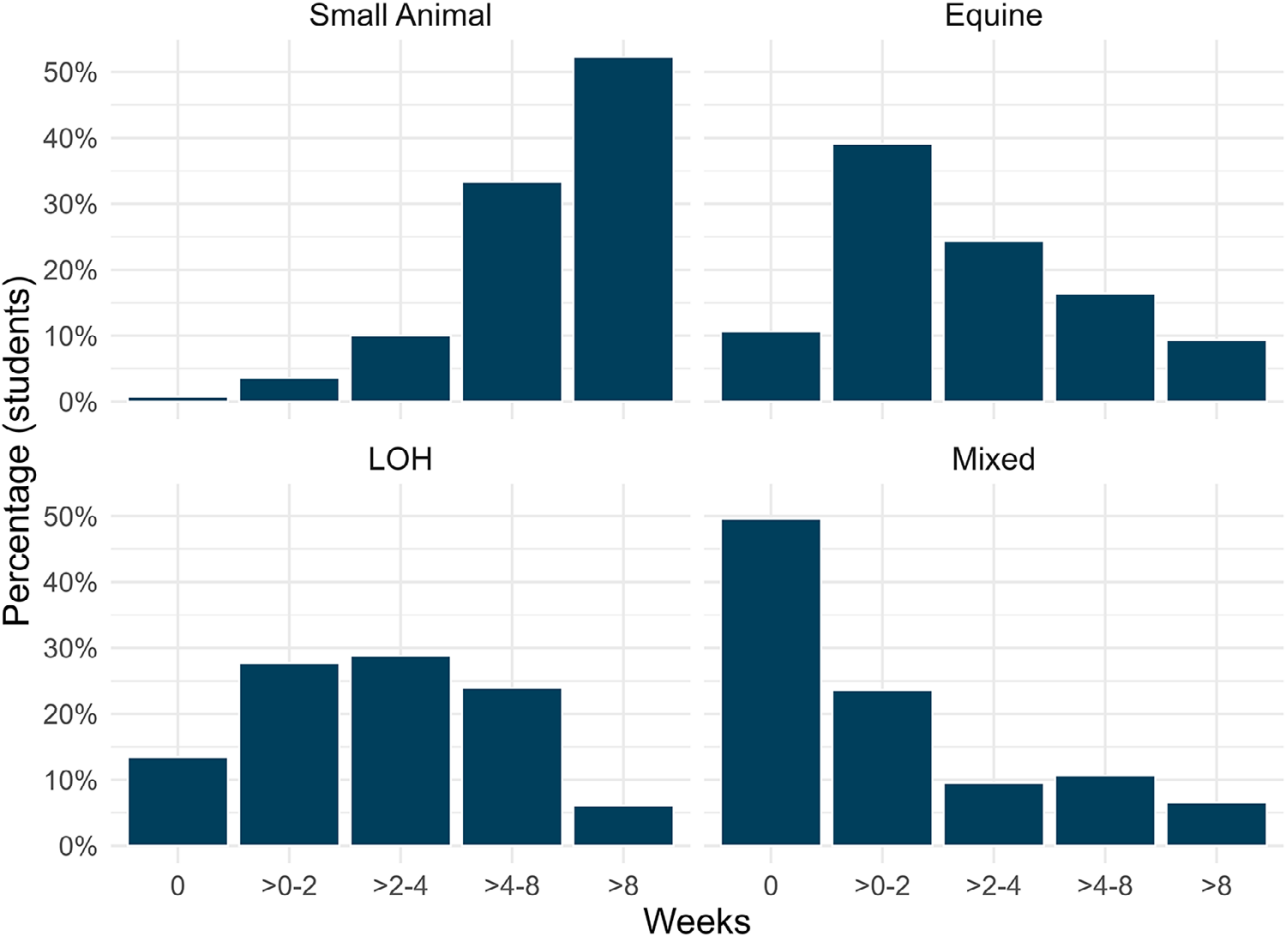
Percentage of veterinary students completing different numbers of weeks of small animal, equine, livestock and One Health (LOH), and mixed extramural studies placements.

Between 121 and 134 students took their final‐year examinations each year from 2020 to 2024. The distribution of examination marks is shown in Figure 2. The mean mark in each examination was 64.3% (standard deviation [SD] 8.0) for LOH, 67.0% (SD 8.9) for equine and 64.7% (SD 9.5) for small animal.

**FIGURE 2:**
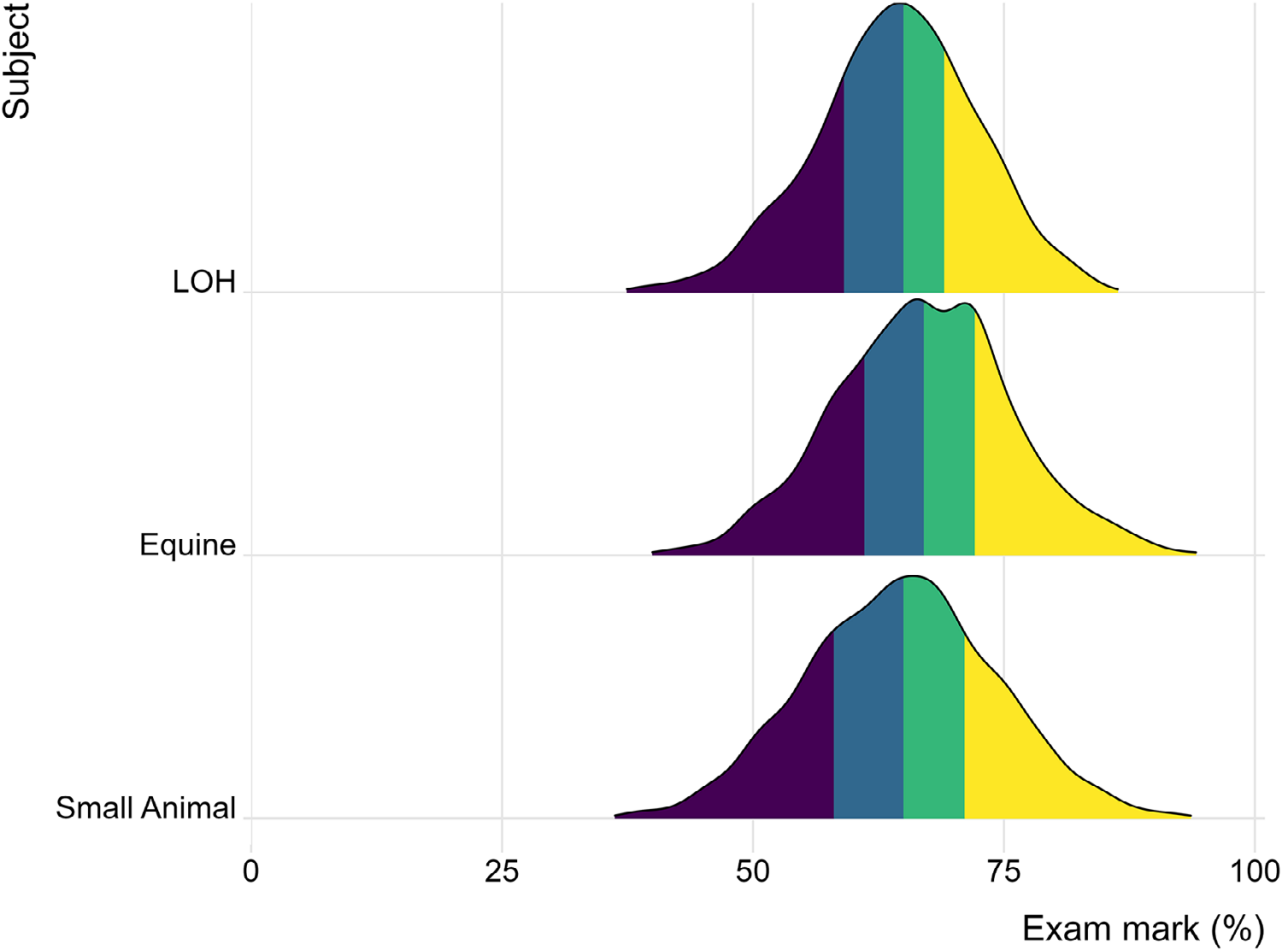
Distribution of marks in the livestock and One Health (LOH), equine and small animal final veterinary examinations. Shading indicates quartile boundaries within each subject.

### Outcome 1: Examination mark

#### LOH examination

There was no evidence that the slope for LOH EMS weeks differed from the slope for mixed EMS weeks (*p*  =  0.15). Therefore, the simpler model combining weeks of LOH and mixed EMS was used. A significant interaction between LOH/mixed EMS weeks and average preclinical examination mark was retained (*p* = 0.01).

In the final model for LOH examination marks (Table 1, adjusted *R* ^2^ = 0.364), the number of LOH/mixed EMS weeks was positively associated with performance (*β* = 0.48, 95% confidence interval [CI]: 0.30‒0.66, *p* < 0.01). A significant interaction between LOH/mixed EMS weeks and average preclinical mark (*β* = ‒0.02, 95% CI: ‒0.04 to ‒0.01, *p* = 0.01) indicated that the positive association between EMS weeks and examination mark was greater among students with lower average preclinical examination performance. The interaction between LOH/mixed EMS weeks and average preclinical mark is illustrated in Figure 3. The average preclinical mark and year of examination (years 2021 and 2022 compared with 2020) were also independently associated with the LOH examination mark.

**TABLE 1:** Estimated regression parameters, standard errors (SE), *t*‐values and *p*‐values for the linear regression model of livestock and One Health (LOH) examination mark (*n* = 639).

Term	Estimate	SE	*t*‐Value	*p*‐Value
Intercept	60.07	2.07	29.04	<0.01
Weeks of LOH/mixed EMS	0.48	0.09	5.35	<0.01
Weeks of equine EMS	0.03	0.10	0.31	0.76
Weeks of small animal EMS	0.03	0.09	0.28	0.78
Average preclinical examination mark	0.87	0.07	12.40	<0.01
2020 (Ref.)				
2021	2.11	1.04	2.02	0.04
2022	2.37	1.07	2.21	0.03
2023	1.05	1.04	1.02	0.31
2024	0.33	0.87	0.37	0.71
Weeks of LOH/mixed EMS × average preclinical examination mark	‒0.02	0.01	‒2.46	0.01

*Note*: Adjusted *R* ^2^ = 0.364.

Abbreviation: EMS, extramural studies.

**FIGURE 3:**
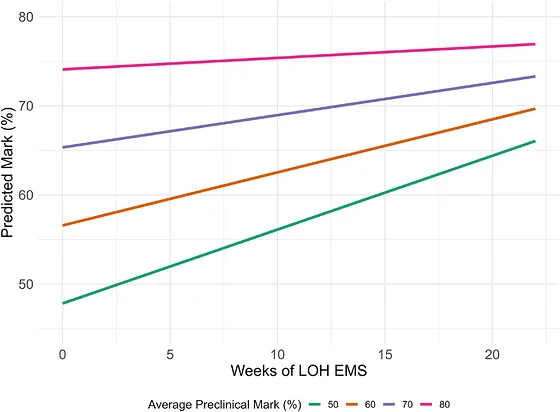
Model‐predicted livestock and One Health (LOH) examination mark by the number of weeks of LOH clinical extramural studies (EMS), stratified by average preclinical examination mark. Predictions represent marginal effects, averaged across the year of examination.

#### Equine examination

The slope for equine EMS weeks differed from the slope for mixed EMS weeks (*p*  =  0.049). Therefore, the model with separate equine and mixed EMS terms was used. A significant interaction between equine EMS weeks and year of examination was retained (*p* = 0.03).

In the final equine model (Table 2, adjusted *R* ^2^ = 0.419), equine EMS weeks were positively associated with examination marks (*β* = 0.89, 95% CI: 0.53‒1.25, *p* < 0.01). Year of examination was independently associated with examination marks, with students in 2022 (*β* = 3.58, *p* = 0.02) and 2024 (*β* = 7.05, *p* < 0.01) scoring significantly higher than those in 2020. Interaction terms indicated that the equine EMS effect was weaker in 2021 (*β* = ‒0.74, 95% CI: ‒1.33 to ‒0.15, *p* = 0.01), 2022 (*β* = ‒0.66, 95% CI: ‒1.13 to ‒0.19, *p* = 0.01) and 2024 (*β* = ‒0.61, 95% CI: ‒1.11 to ‒0.10, *p* = 0.02) compared with 2020, while the 2023 interaction was not significant. The interaction between equine EMS weeks and examination year is illustrated in Figure 4. Mixed EMS showed a weaker positive association with examination marks (*β* = 0.22, 95% CI: 0.00‒0.44, *p* = 0.046). However, this borderline result should be interpreted cautiously. This effect was not moderated by year. The average preclinical mark was independently associated with the equine examination mark.

**TABLE 2:** Estimated regression parameters, standard errors (SE), *t*‐values and *p*‐values for the linear regression model of equine examination mark (*n* = 639).

Term	Estimate	SE	*t*‐Value	*p*‐Value
Intercept	61.63	2.35	26.20	<0.01
Weeks of equine EMS	0.89	0.18	4.87	<0.01
Weeks of LOH EMS	0.03	0.12	0.28	0.78
Weeks of small animal EMS	0.07	0.10	0.75	0.45
Weeks of mixed EMS	0.22	0.11	2.00	0.05
Average preclinical examination mark	0.79	0.05	16.92	<0.01
2020 (Ref.)				
2021	1.84	1.59	1.16	0.25
2022	3.58	1.53	2.34	0.02
2023	‒0.51	1.55	‒0.33	0.74
2024	7.05	1.43	4.93	<0.01
Weeks of equine EMS × 2021	‒0.74	0.30	‒2.47	0.01
Weeks of equine EMS × 2022	‒0.66	0.24	‒2.75	<0.01
Weeks of equine EMS × 2023	‒0.45	0.28	‒1.62	0.11
Weeks of equine EMS × 2024	‒0.61	0.26	‒2.36	0.02

*Note*: Adjusted *R* ^2^ = 0.419.

Abbreviations: EMS, extramural studies; LOH, livestock and One Health.

**FIGURE 4:**
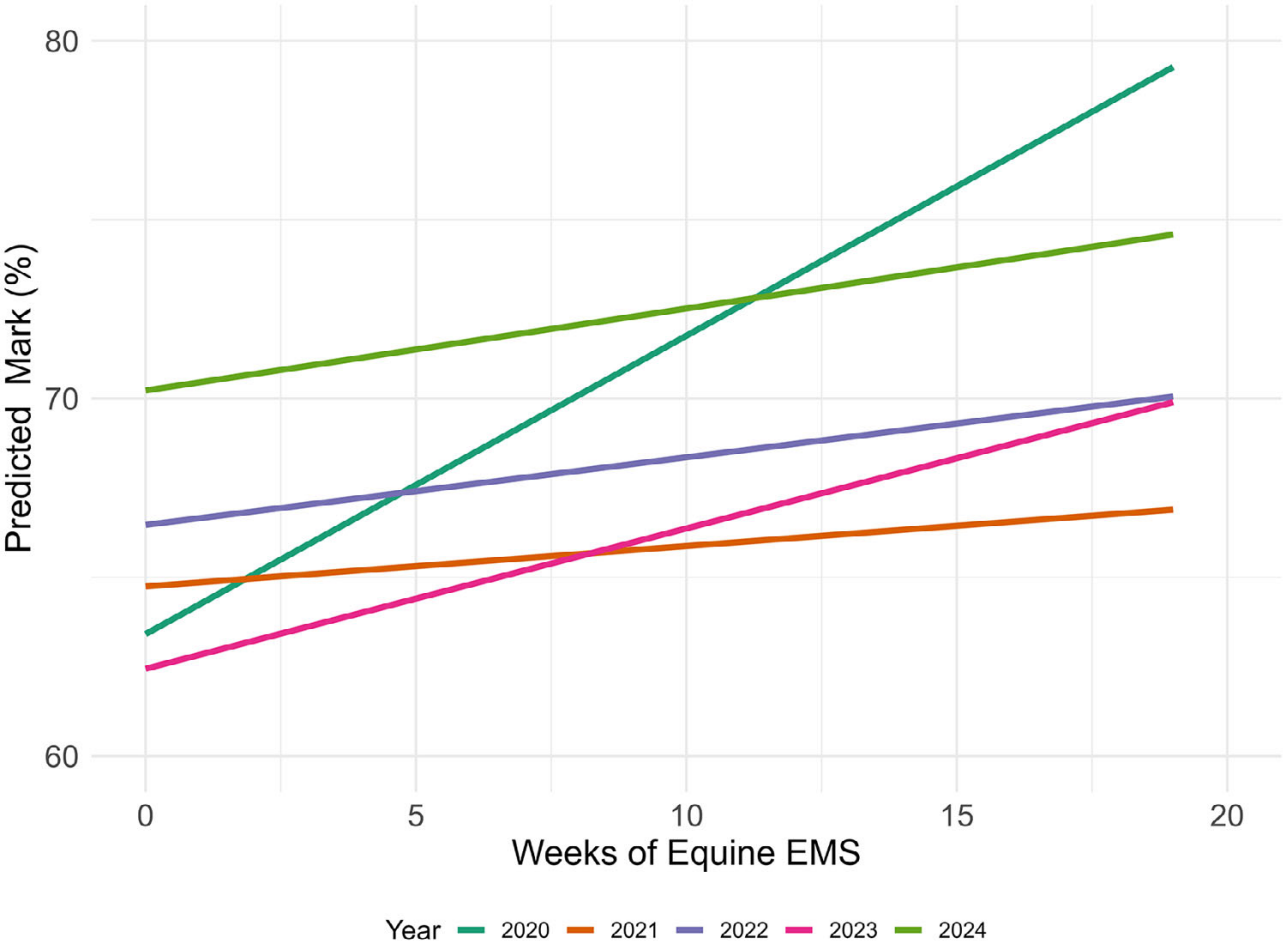
Model‐predicted equine examination mark by the number of weeks of equine clinical extramural studies (EMS), stratified by year of examination. Predictions represent marginal effects, averaged across average preclinical examination marks and weeks of mixed EMS.

#### Small animal examination

There was no evidence that the slope for small animal EMS weeks differed from the slope for mixed EMS weeks (*p*  =  0.76), with similar adjusted *R* ^2^ values (0.425 vs. 0.426). Therefore, the model combining weeks of small animal and mixed EMS was used. No interaction terms were statistically significant and were excluded from the final model.

In the final small animal model (Table 3, adjusted *R* ^2^ = 0.426), the number of small animal/mixed EMS weeks was positively associated with examination performance (*β* = 0.37, 95% CI: 0.18‒0.57, *p* < 0.01). No significant interactions were observed. The average preclinical mark was associated with final‐year performance. Students taking the examination in 2024 scored significantly lower than those who took it in 2020 (*β* = ‒2.12, 95% CI: ‒4.04 to ‒0.20, *p* = 0.03).

**TABLE 3:** Estimated regression parameters, standard errors (SE), *t*‐values and *p*‐values for the linear regression model small animal examination mark (*n* = 639).

Term	Estimate	SE	*t*‐Value	*p*‐Value
Intercept	59.75	2.31	25.88	<0.01
Weeks of small animal/mixed EMS	0.37	0.10	3.73	<0.01
Weeks of LOH EMS	0.19	0.13	1.50	0.13
Weeks of equine EMS	‒0.02	0.12	‒0.21	0.83
Average preclinical examination mark	1.00	0.05	20.25	<0.01
2020 (Ref.)				
2021	0.57	1.17	0.49	0.63
2022	2.45	1.21	2.03	0.04
2023	‒0.35	1.17	‒0.30	0.76
2024	‒2.12	0.98	‒2.16	0.03

*Note*: Adjusted *R* ^2^ = 0.426.

Abbreviations: EMS, extramural studies; LOH, livestock and One Health.

### Outcome 2: Examination success

Across all the models, associations between species‐specific EMS weeks and the likelihood of passing the corresponding examination were weaker and less consistent than those observed for examination marks, likely reflecting limited statistical power due to low failure rates.

For the small animal examination (28/639 failures), each additional week of small animal EMS was associated with 12% higher odds of passing (OR 1.12, 95% CI: 1.02‒1.25, *p* = 0.02). For the LOH examination (20/639 failures), each additional week of LOH EMS was associated with 21% higher odds of passing (OR 1.21, 95% CI: 0.99‒1.56, *p* = 0.07), although this did not reach statistical significance. For the equine examination, additional equine EMS weeks were associated with a non‐significant decrease in the odds of passing (OR 0.88, 95% CI: 0.76‒1.05, *p* = 0.13), which contrasts with the positive association observed for examination marks. This apparent discrepancy likely reflects the very low failure rate (10/639 students); with so few failures, logistic regression estimates become unstable and highly sensitive to the characteristics of the small number of students who failed. The wide confidence interval underscores the uncertainty of this estimate and limits meaningful interpretation.

Across all three models, higher average preclinical marks were consistently associated with increased odds of passing. No significant associations with year of examination were observed.

### Outcome 3: Overall examination success

Among students with an average preclinical mark below 60% (*n* = 106; Figure 5), 71.4% (35/49) of those who had fewer than 2 weeks of EMS in any of small animal, equine or LOH passed all their final‐year examinations, compared to 87.7% (50/57) of those who completed at least 2 weeks in each discipline. This difference was borderline statistically significant (Fisher's exact test, *p* = 0.05), with estimated odds of passing all examinations approximately 2.83 times higher among students with a balanced clinical EMS experience (95% CI: 0.95–9.19).

**FIGURE 5:**
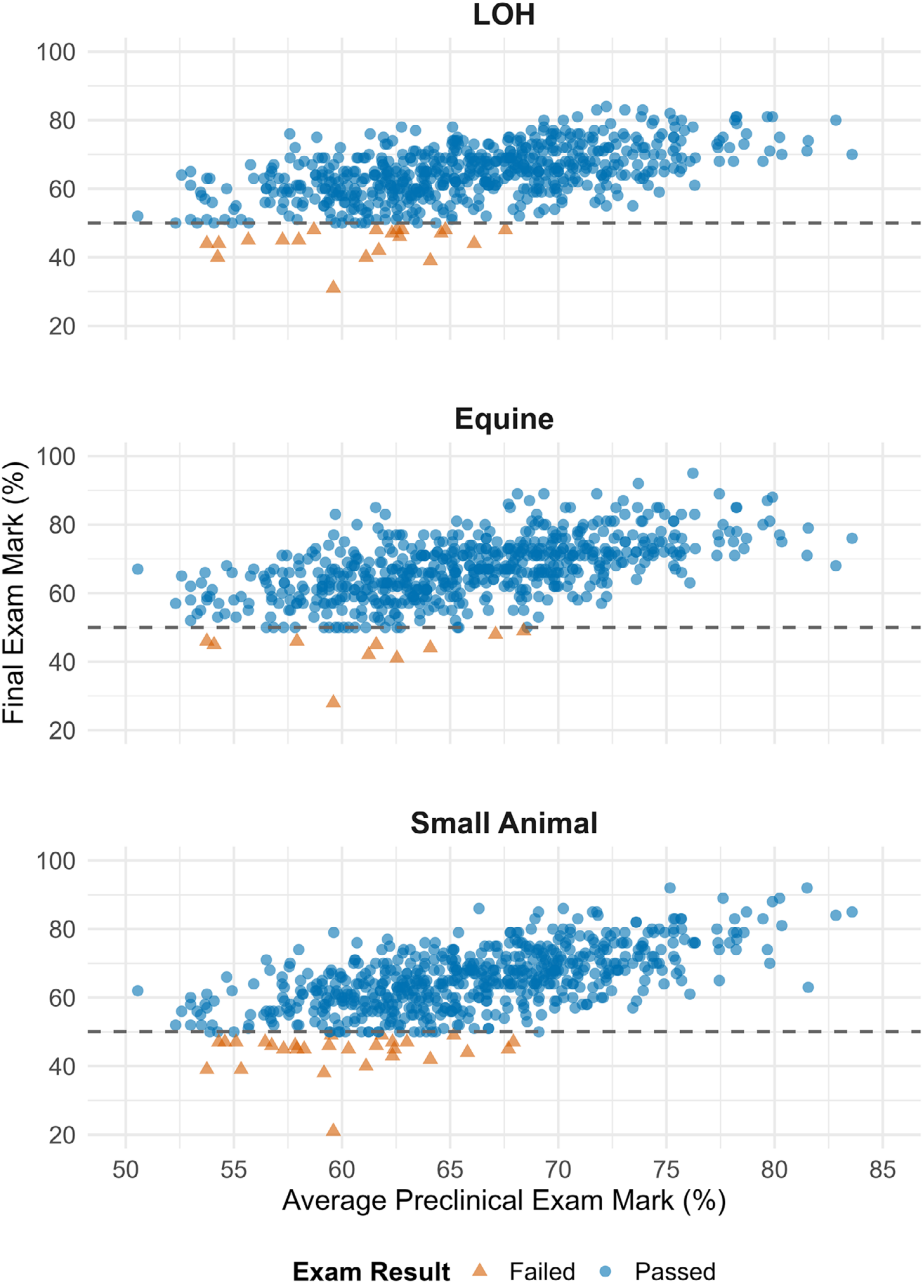
Average preclinical examination marks in relation to performance in the final livestock and One Health (LOH), equine and small animal examinations. Each point represents an individual student (*n* = 639). The points are coloured and shaped by examination result (passed vs. failed), with a dashed line indicating the 50% pass mark.

When mixed EMS was regarded as an alternative way to achieve a balanced EMS experience, two students were re‐labelled as having balanced EMS (≥8 weeks mixed EMS), resulting in 70.2% (33/47) of students who had fewer than 2 weeks in any species‐specific EMS category or fewer than 8 weeks of mixed EMS passing all examinations, compared to 88.1% (52/59) of those who met either criterion. This difference was statistically significant (Fisher's exact test, *p* = 0.03), with estimated odds of passing all examinations approximately 3.12 times higher in the group meeting this balanced EMS definition (95% CI: 1.05–10.14). When a lower threshold of 6 weeks of mixed EMS was used, the difference in examination success rates was smaller (72.5% vs. 84.8%) and not statistically significant (Fisher's exact test, *p* = 0.138).

## DISCUSSION

This study examined associations between the number and category of clinical EMS placements and final‐year examination performance over five cohorts of students. Across examination marks, pass/fail outcomes and overall pass rates, there was evidence that increased EMS was positively associated with examination performance, particularly when placements aligned with the examination subject area. However, these results should not be interpreted as evidence of causality. As discussed below, the observational study design means that unmeasured factors may explain part or all of the observed associations.

For LOH and small animal examinations, more weeks of relevant EMS, including mixed EMS, were associated with higher marks, with the effect particularly pronounced among students with lower preclinical academic performance. One interpretation is that hands‐on clinical experience reinforces learning for students who find lecture‐based preclinical teaching less effective; for example, engaging with real‐life cases can provide additional context for otherwise more abstract ideas that may be challenging to grasp and retain.^13^ This aligns with experiential learning theory and deliberate practice frameworks^14^, ^15^; however, these patterns could also reflect student characteristics such as motivation, career focus or conscientiousness that influence both EMS choices and examination performance.^3^, ^16^, ^17^

The link between equine EMS and examination marks varied across different years. This likely reflects the unique position of the 2020 cohort, which completed the majority of its clinical EMS requirements prior to the onset of the COVID‐19 pandemic. Their strong positive association between EMS weeks and examination marks may therefore represent a ‘pre‐pandemic baseline’ for the educational value of placements. In contrast, for subsequent cohorts (2021‒2024), the association was weaker. This could be attributed to a ‘dilution’ of the placement experience during the pandemic; even when students could secure placements, strict social distancing measures may have restricted their involvement in consultations or prevented students from travelling in vehicles with veterinarians. However, it is unclear why the moderating effect of year was observed only with the equine examination, given that these external factors were consistent across all subjects.

In all the models, preclinical academic performance was the most significant predictor of examination scores, which is consistent with previous veterinary education research showing that prior academic achievement predicts later success.^18^

There was also weaker evidence that relevant EMS was associated with a higher likelihood of passing the corresponding examination. However, low overall failure rates, particularly in equine, limited the statistical power of this analysis. In an exploratory analysis of students with preclinical marks below 60%, a ‘balanced’ EMS experience was associated with higher odds of passing all three examinations, but confidence intervals were wide, reflecting the small sample size in this subset. These findings should be considered hypothesis‐generating and require confirmation in larger samples. This result may reflect the advantages of broader clinical exposure, which would support the university's previous policy requiring students to complete at least 2 weeks of small animal, equine and LOH EMS. Alternatively, as most practising veterinarians work in small animal practice, and career aspirations are a major driver for EMS choices, proactively seeking varied EMS as a student may reflect student personality traits that are also linked to better academic performance.^3^, ^19^ For example, autonomous motivation has previously been associated with improved academic outcomes.^20^, ^21^ Further research on UK veterinary students would be beneficial for gaining a deeper understanding of the factors that influence students’ planning and priorities for EMS.

There are several limitations to this study. The categorisation of EMS was self‐reported, and classification, especially for mixed EMS, might reflect practice setting rather than actual case exposure, potentially leading to misclassification bias. Mixed EMS might include a variable distribution of cases across the three disciplines (i.e., small animal, equine and LOH), making it difficult to interpret these specific results. Additionally, there are differences in the quality of different EMS placements and in the level of engagement students have during placements.^22^ Therefore, each week of EMS does not provide an equal learning experience, which could potentially weaken the statistical association between EMS and examination performance. Other data would have been interesting to include in the analysis. It was unknown whether students had extenuating circumstances that may have influenced both examination performance and possibly EMS opportunities. Other factors that are likely to influence examination performance, such as attendance records or engagement with virtual learning resources during their final year, would have been interesting covariates to include in the analysis. To investigate whether there is a causative association between EMS and examination performance, future work could incorporate more detailed recording of EMS content and underlying student characteristics.

Clinical EMS placements likely develop competencies beyond those assessed in written examinations, including communication, teamwork, clinical reasoning and professional behaviour. Practical examinations at the University of Liverpool underwent substantial structural changes during the study period, transitioning from objective structured clinical examinations to various iterations of workplace‐based assessment, with no practical examinations during COVID‐19‐affected years. This variability precluded meaningful analysis of practical examination outcomes in relation to EMS. Future research incorporating standardised practical assessments or workplace‐based assessments could provide additional insights into the broader association between EMS and student end‐of‐programme success.

The effects of COVID‐19 disruptions and changes to RCVS policy regarding species‐specific EMS requirements are also likely to have influenced the observed associations. It will be of interest to evaluate the effects of the 2024 RCVS guidelines, under which all students must now complete 10 weeks of animal husbandry EMS and 20 weeks of clinical EMS with no species‐specific requirements.

Overall, our results suggest that for veterinary students at the University of Liverpool, EMS breadth and relevance may be linked to, but not necessarily cause, better final‐year examination performance. Academic advisers could encourage greater breadth in EMS placements, especially for students with weaker prior examination performance, while being mindful that placement availability and accessibility disproportionately affect lower‐resourced students.^3^

## CONCLUSIONS

This study found positive associations between clinical EMS and final‐year examination results, particularly for examination marks. However, these findings do not prove direct or causal effects of EMS. It is just as likely, if not more so, that EMS choices reflect student behaviours and traits that also affect examination performance. Nevertheless, supporting students to undertake varied EMS across all disciplines may be a practical strategy to potentially improve academic results.

## AUTHOR CONTRIBUTIONS

*Conceptualisation, methodology, formal analysis, data curation and writing—original draft*: Matthew Barden. *Conceptualisation, methodology and writing—review and editing*: Emma Ormandy. *Conceptualisation and writing—review and editing*: Melanie Chapman and Alistair Freeman. *Conceptualisation, methodology, data curation and writing—review and editing*: Kieron Salmon.

## CONFLICT OF INTEREST STATEMENT

The authors declare they have no conflicts of interest.

## FUNDING INFORMATION

The authors received no specific funding for this work.

## ETHICS STATEMENT

Ethical approval for research projects conducted as part of this programme was granted by the University of Liverpool Professional Services Research Ethics Committee (REF 14008).

## Data Availability

The data that support the findings of this study are available from the corresponding author upon reasonable request.
